# Stability of Multicomponent Antidote Parenteral Formulations for Autoinjectors against Chemical War Agents (Neurotoxics)

**DOI:** 10.3390/pharmaceutics16060820

**Published:** 2024-06-17

**Authors:** María José Rodríguez Fernández, Daniel Hernández, Brayan Javier Anaya, Dolores R. Serrano, Juan José Torrado

**Affiliations:** 1 Defense Military Pharmacy Center (CEMILFARDEF), Colmenar Viejo, 28770 Madrid, Spain; 2 Quick Identification Laboratory, Military Emergency Unit, Torrejón de Ardoz, 28850 Madrid, Spain; 3 Department of Pharmaceutics, School of Pharmacy, Complutense University of Madrid, 28040 Madrid, Spain; dhfranco25@gmail.com (D.H.); branaya@ucm.es (B.J.A.); 4 Institute of Industrial Pharmacy, Complutense University of Madrid, 28040 Madrid, Spain

**Keywords:** pralidoxime, obidoxime, atropine, midazolam, elastomer, stability, primary packing material

## Abstract

Combinations of different drugs are formulated in autoinjectors for parenteral administration against neurotoxic war agents. In this work, the effects on the chemical stability of the following three variables were studied: (i) type of drug combination (pralidoxime, atropine, and midazolam versus obidoxime, atropine, and midazolam); (ii) pH (3 versus 4); and (iii) type of elastomeric sealing material (PH 701/50 C BLACK versus 4023/50 GRAY). Syringes were stored at three different temperatures: 4, 25, and 40 °C. Samples were assayed at different time points to study the physical appearance, drug sorption on the sealing elastomeric materials, and drug content in solution. Midazolam was unstable in all tested experimental conditions. Drug adsorption was observed in both types of sealing elastomeric materials and was significantly (*p* < 0.01) dependent on the lipophilicity of the drug. The most stable formulation was the combination of pralidoxime and atropine at pH 4 with the elastomeric sealing material 4023/50 GRAY.

## 1. Introduction

In recent years, the possibility of exposure to chemical warfare agents, particularly nerve agents, has occurred in military operations such as the well-known attacks in Syria in 2013 [1], in the civil sphere such as the Navalni case in 2020 [2], or in accidental exposure such as the bottle of cologne in a park in Amesbury (United Kingdom) in 2018 [3].

Any Chemical, Biological, Radiological, and Nuclear (CBRN) incident causes great panic and chaos in the population, sometimes causing a great collapse in health systems. For this reason, the need to develop effective health countermeasures, pharmacological therapies that counteract the undesirable effects caused by neurotoxic agents, is accentuated.

Treatment against organophosphate poisoning has been the subject of study for decades. The focus in developing effective treatments has been on highly toxic chemical warfare agents (CWAs) such as sarin, tabun, soman, VX, and Novichok, presenting, among them, high variability in their physicochemical, toxicodynamic, and toxicokinetic properties. Even small doses of these substances can cause clinical poisoning, characterized by muscarinic-like, nicotinic-like, and central nervous system symptoms. These symptoms develop rapidly, necessitating prompt and appropriate treatment, often including intensive care. The critical importance of immediate antidote administration is underscored by the short half-life of the ‘aging’ process of the enzyme–organophosphorus compound complex. For example, the half-life for the acetylcholinesterase (AChE)–soman complex is only 3.5 min, and for the AChE–sarin complex, it is approximately 18 min. After these periods, only 50% of the inhibited enzyme can be reactivated [4].

An autoinjector is a user-friendly device intended for the intramuscular administration that can be utilized for self-aid or buddy-aid on the battlefield when needed. The use of these autoinjectors to administer drugs (antidotes) can save the lives and health of soldiers before they receive specialized medical care, such as in a hospital [4].

The most effective therapy for years has been based on an antimuscarinic component such as atropine, a natural antagonist of acetylcholine, to decrease the cholinergic effects, used in conjunction with the administration of an oxime that is nucleophilic enough to reactivate acetylcholinesterase, such as pralidoxime, obidoxime, or HI-6, including a third compound from the group of benzodiazepines to counteract seizures on the central nervous system, such as diazepam or midazolam.

In Spain, since the 1990s, autoinjector systems have been manufactured in military–industrial settings containing atropine and oxime, but in 2015, the portfolio of autoinjectors was expanded, with a new one for benzodiazepines; both autoinjectors should be administered in the case of acute poisoning by neurotoxic agents. In other countries, the attempt to develop multicomponent autoinjectors with three active ingredients in the same dosage form has been explored, making the administration of all drugs required easier [5,6,7,8]. The main differences are the type of drug within the same chemical family, based on the military health doctrine of each country, and on the stability characteristics of the active principles in the pharmaceutical form.

Although the chemical stability of monocomponent parenteral formulations containing pralidoxime [9,10], obidoxime [11], atropine [12], and midazolam [13,14] has been reported as stable, the combination of many of them in the same autoinjector formulation has never been explored and may lead to physicochemical interactions and a loss of stability. Apart from drug chemical stability in solution, another crucial point to identify is the drug adsorption within the elastomeric gasket materials used to manufacture the autoinjectors. Higher adsorption rates can detrimentally reduce the amount of drug available in a solution that is administered to the patient. In a previous work, based on benzodiazepine autoinjector formulations, diazepam demonstrated a more stable profile compared to midazolam but 2-fold greater adsorption on the elastomeric gaskets [14]. This suggested that midazolam may be a better candidate for multicomponent autoinjector formulations. Also, the safety and pharmacokinetic parameters of midazolam administered with an autoinjector evidenced that no serious adverse events occurred in a range from 5 to 30 mg [15]. 

The main aim of this work was to study the chemical stability of two multicomponent formulations against neurotoxic agents (F1: atropine sulfate, pralidoxime chloride, and midazolam chloride; F2: atropine sulfate, obidoxime chloride, and midazolam chloride), considering several fabrication variables: the sealing elastomeric joints of the primary packaging (chlorobutyl made—PH 701/50 BLACK seals—coded as JEA and newer developed materials based on bromobutyl 4023/50 GRAY—coded as JEN), pH of the solution (3 and 4), and storage temperature (4 °C for 12 months, 25 °C for 53 months and 40 °C for 9 months). The adsorption of the drugs in the sealing gaskets was also studied. In a previous work [14], the bromobutyl—4023/50 GRAY sealing gasket proved to be a better option than older materials such as chlorobutyl—PH 701/50 BLACK, especially for midazolam monocomponent formulations. In this work, the compatibility of this new sealing gasket with pralidoxime, obidoxime, and atropine was also studied.

## 2. Materials and Methods

### 2.1. Materials 

Active Pharmaceutical Ingredients (APIs) and excipients for formulations were of pharmaceutical grade. Pralidoxime hydrochloride was supplied by Raschig GmbH (Ludwigshafeb, Germany), obidoxime hydrochloride by Merck KGaA (Darmstadt, Germany), atropine sulfate by Shaoxing Minsheng Pharmaceutical Co. (Shaoxing city, China), midazolam hydrochloride by Fagron Iberica (ES) (Terrassa, Barcelona, Spain), sodium metabisulphite and hydrochloric acid 37% by Panreac Química S.L.U. (Castellar del Valles, Barcelona, Spain). Glass syringes were purchased from AGRADO, S.L. (Valdemoro, Madrid, Spain). The sealing materials, chlorobutyl PH 701/50 C BLACK, and bromobutyl 4023/50 GRAY gaskets were purchased from West Pharmaceutical Services Inc. (Exton, PA, USA). All reagents were of analytical grade and were used as supplied by Fisher Scientific (Madrid, Spain).

### 2.2. Preparation of Formulations Loaded into Autoinjectors

Parenteral formulations were elaborated according to cGMP in class A, B, and C cleanroom areas of the pharmaceutical laboratory of the Spanish Army (CEMILFARDEF) in Colmenar Viejo in Madrid (Spain). Batch formulations were of 900 mL quantity, enough to fill 114 syringes for each drug solution.

Formulation 1 (F1) was elaborated by dissolving 0.7 g of sodium metabisulphite in water for injection followed by 180 g of pralidoxime hydrochloride, 3 g of midazolam hydrochloride, and 0.6 g of atropine sulphate. Hydrochloric acid 37% was added to adjust pH either to 3.0 ± 0.1 or 4.0 ± 0.1. Finally, water for injection was added up to 900 mL and filtered through a 0.2 µm sterile filter (Supor^®^, PALL technologies, Port Washington, NY, USA). Formulation 2 (F2) was prepared in a similar way to formulation 1, but the 180.0 g of pralidoxime hydrochloride was replaced with 66.0 g of obidoxime hydrochloride. The liquid formulations were packed in 3 mL cartridge syringe glass Type I (Schott, Schott Pharma, Mainz, Germany) with two different sealing materials described previously [14].

### 2.3. Stability Study and Specification Limits 

ICH Q1A(R2) guidelines were applied for the stability study [16]. The characteristics of the storage and sampling conditions are shown in Table 1. Sampling and assays were performed in triplicate and included an evaluation of the physical appearance of the liquid contained in the syringe, the possible presence of crystals, the appearance of the sealing elastomeric material, API concentration in the liquid contained in the glass syringe, and amount of API adsorbed in the elastomeric sealing material.

The following quality parameters were evaluated as indicators for the stability of the autoinjectors: (i) the specification limits for the API concentration in the syringe was 100 ± 10% for pralidoxime hydrochlorhydrate, obidoxime hydrochlorhydrate, and midazolam hydrochlorhydrate while the limits for atropine sulphate were 100 ± 7%; (ii) the amount of API adsorbed in the sealing elastomeric material with a limit below 1% API content per syringe; and (iii) the physical appearance of the liquid content and the sealing elastomeric material. The liquid content of the syringe must be pale yellow and transparent for formulation 1 and orange transparent for formulation 2, without particles in any of them. The sealing should have no signs of defects and must retain the initial color (black or grey, respectively) and its flexibility.

### 2.4. Quantification of APIs in Solution and Adsorbed on the Elastomeric Sealing Materials by HPLC

**Figure 1:**
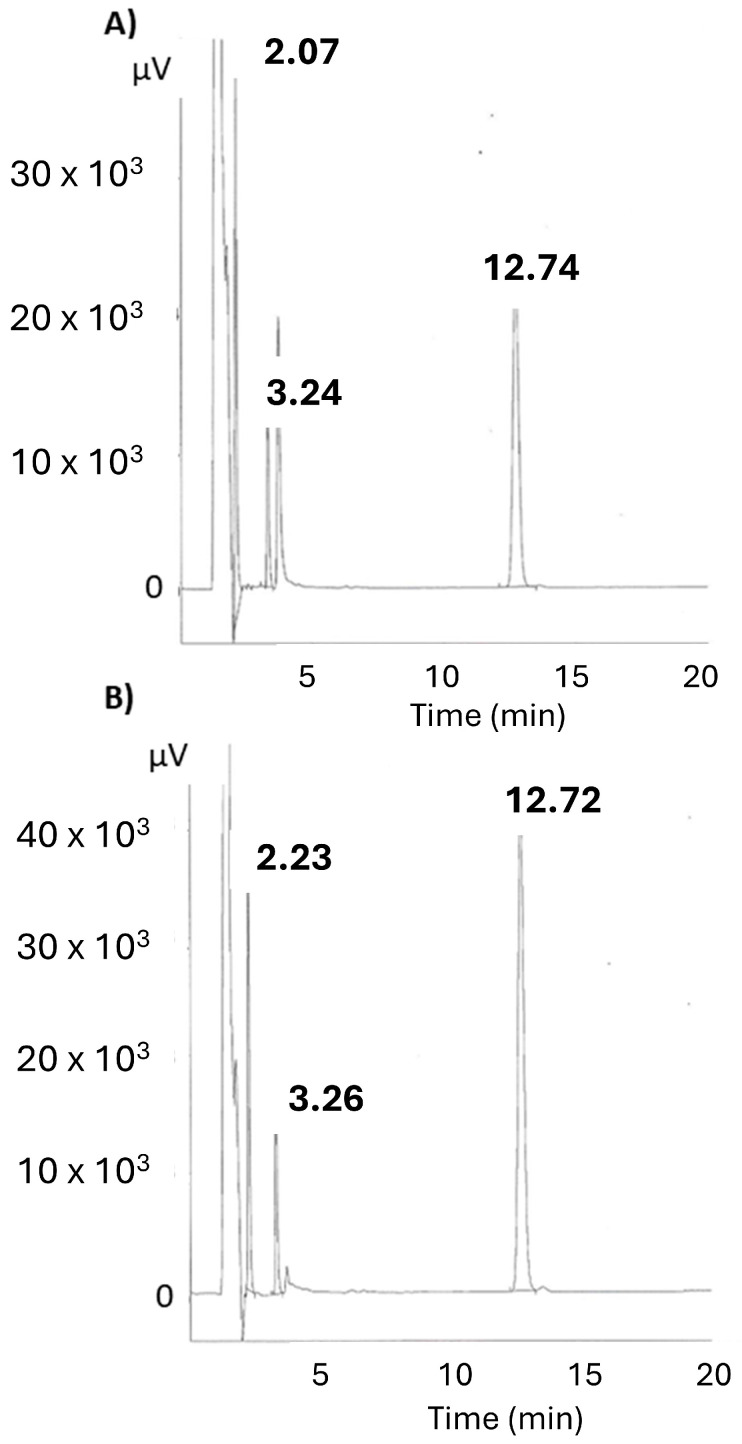
Chromatograms of API components of formulations 1 (**A**) and 2 (**B**). Retention times (minutes) of APIs were 2.1 min for pralidoxime, 2.2 for obidoxime, 3.3 for atropine, and 12.7 for midazolam.

Modular Jasco (Jasco Inc, Tokyo, Japan) HPLC equipment with a Jasco PU-1580 pump, a Jasco AS-2050-Plus autosampler fitted to a 100 μL sampling loop, and a UV-visible detector Jasco UV-1575 were used. The wavelength detection was set at 220 nm. The mobile phase was based on the HPLC method described in USP 38 (2015) [17] for atropine assay, consisting of a mixture of acetonitrile (HPLC gradient grade)/aqueous phase at 30:70 (*v*:*v*) proportions. The aqueous phase was composed of a buffer solution of 1.8 g/L KH_2_PO_4_ with 2.5 g/L of sodium heptane sulfonated acid pH 2.5 adjusted with orthophosphoric acid. The mobile phase was filtered through a 0.45 µm filter (Supor^®^-450, Pall Corporation Ref 60173, Port Washington, NY, USA) and degassed. The flow rate was fixed at 1 mL/min. The stationary phase was a Zorbax Eclipse XDB-C18 column 150 × 4.6 mm with a particle size of 3.5 µm. The injection volume was 20 µL. Test samples (0.1 mL) of the liquid formulations were diluted with 100 mL of a mixture of methanol (HPLC gradient grade)/purified water (50:50) (*v*/*v*) and then filtered through a Millex^®^ HV PVDF Millipore filter of 0.45 μm. Figure 1 shows the chromatogram acquired for formulations 1 and 2. Retention times (RT) for pralidoxime, obidoxime, atropine, and midazolam were 2.1, 2.2, 3.3, and 12.7 min, respectively. In our experimental conditions, the retention time for the solvent front was the same as the dead time determined with uracil: both were 1.4 min.

Linearity was studied between 5 and 125% of the theoretical API values. Correlation coefficients were all at least 0.99 for all studied APIs. Repeatability and reproducibility were good, with RSD values lower than 5% for all studied APIs. Typical slopes for pralidoxime, obidoxime, atropine, and midazolam were 4339.4, 2006.5, 5.2, and 163.4, respectively. Limits of detection for pralidoxime, obidoxime, atropine, and midazolam were 2.2, 3.6, 11.7, and 3.9 ng/mL, respectively. Limits of quantification for pralidoxime, obidoxime, atropine, and midazolam were 10, 3.66, 0.03, and 0.16 µg/mL, respectively.

The amount of API adsorbed on the sealing elastomeric gaskets was also quantified using a previously described method [14]. The two gaskets from each syringe were dried with tissue paper, soaked in a closed glass container with a mixture of 20 mL of methanol (HPLC gradient grade)/purified water (50:50) (*v*/*v*), mixed in a vortex, sonicated in an ultrasonic bath of 720 W and 50/60 Hz (J.P. Selecta ref. 3000838, Madrid, Spain) for 15 min, and then left under constant stirring for 48 h. Afterward, the supernatant was filtered and the API was quantified by HPLC in the same conditions previously described but with an injection volume of 100 µL.

### 2.5. Relationship between Adsorption of API in the Sealing Primary Package and API Lipophilicity 

Samples stored at 25 ± 2 °C for 53 months were taken. Drug concentration inside the autoinjector and the percentage of API retained in the sealing elastomeric gasket were assayed by the reverse-phase HPLC method described in the previous section. The lipophilicity of the APIs in reverse-phase chromatography can be directly related to the retention times (logRT) of the peaks [18]. 

### 2.6. Imaging 

The surface of the elastomeric sealing at time 0 and after 53 months of exposition to different formulation solutions was imaged using a 9MP 2−200x digital microscope (Conrad Electronics, Wemberg-Köblitz, Germany). The software ImageJ v1.46 was used for image analysis. 

### 2.7. Statistical Data Treatment

The degradation of drug components within the formulations stored at 25 ± 2 °C was adjusted to a first-order kinetic to evaluate the degradation constants. The times required for 10% degradation (t_90_) for pralidoxime, obidoxime, and midazolam or 7% degradation (t_93_) for atropine were estimated. The selection of t_90_ or t_93_ depends on the lower specification limits for each API according to USP 38 [17]. The effects of different variables on these t_90_ and t_93_ parameters were evaluated with an experimental design according to a sign criterion, as described in a previous article [19]. The following three variables (and their signs) were studied: (i) type of oxime: pralidoxime (sign −) or obidoxime (sign +); (ii) pH: 3 (sign −) or 4 (sign +); and (iii) type of elastomeric sealing material: PH 701/50 C BLACK (coded as JEA and sign −) and 4023/50 GRAY (coded as JEN and sign +). Student’s two-tailed paired *t*-test and standard error of regression were performed with Excel (Office 365, Microsoft Corporation, Redmond, WA, USA). Minitab software v.16 (Coventry, UK) was used to predict the drug shelf-life from each formulation being intended as the time in which there is 95% confidence that at least 50% of response is within the specification limits. An upper limit and lower limit of 110 and 90% were taken into consideration for pralidoxime, obidoxime, and midazolam, while 107–93% was considered for atropine [20]. 

## 3. Results and Discussion

**Figure 2:**
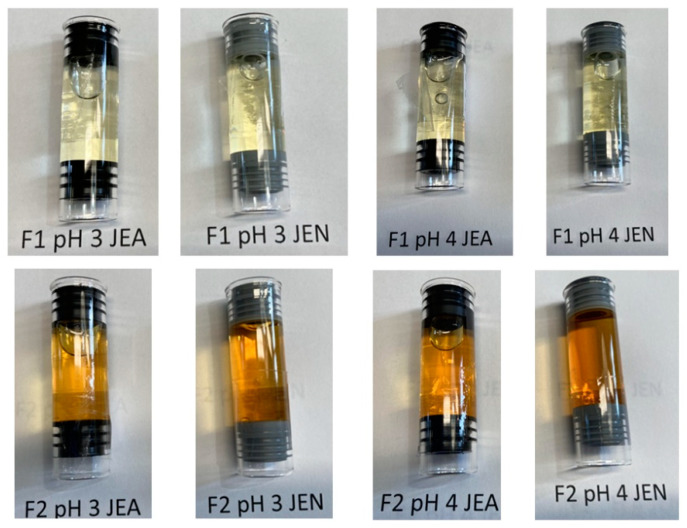
Example of the two different tested formulations (F1—up in the figure and F2—down), different pHs (3 or 4), and different sealing elastomeric gasket pieces (black color—JEA and gray color—JEN) located in the glass syringes of the autoinjectors after 53 months of storage.

Figure 2 shows examples of autoinjectors for formulations 1 and 2 in the two different studied elastomeric gaskets (JEA and JEN) at two different pHs (3 or 4) after 53 months at 25 ± 2 °C. Formulations with obidoxime (F2) exhibited an orange transparent color, while those with pralidoxime (F1) were yellow and transparent. No sign of particles or precipitation was observed in any of the tested samples.

**Figure 3:**
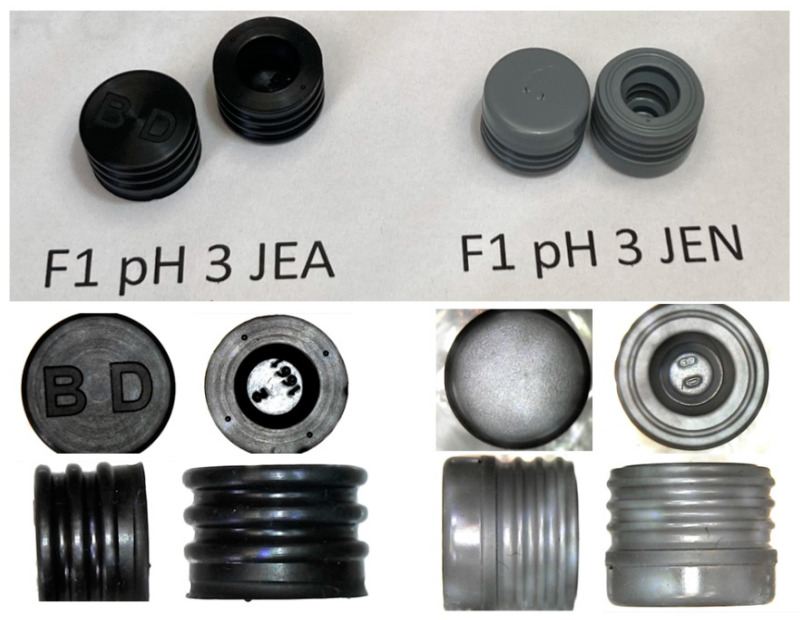
Visual (up) and micrograph (down) aspect of the two different tested sealing gaskets (JEA left and JEN right) located in the glass syringes of formulation 1 pH 3 of the autoinjectors after 53 months of storage at 25 ± 2 °C.

Figure 3 shows the visual appearance of sealing elastomeric pieces after 53 months of storage (top panel). Figure 3 (bottom panel) shows micrographs of representative gaskets after 53 months of storage at 25 ± 2 °C. No visual or micrographic damage was observed in any of the tested gaskets. In terms of the integrity of the elastomeric pieces stored at 25 ± 2 °C, both materials (JEA and JEN) were valid.

**Figure 4:**
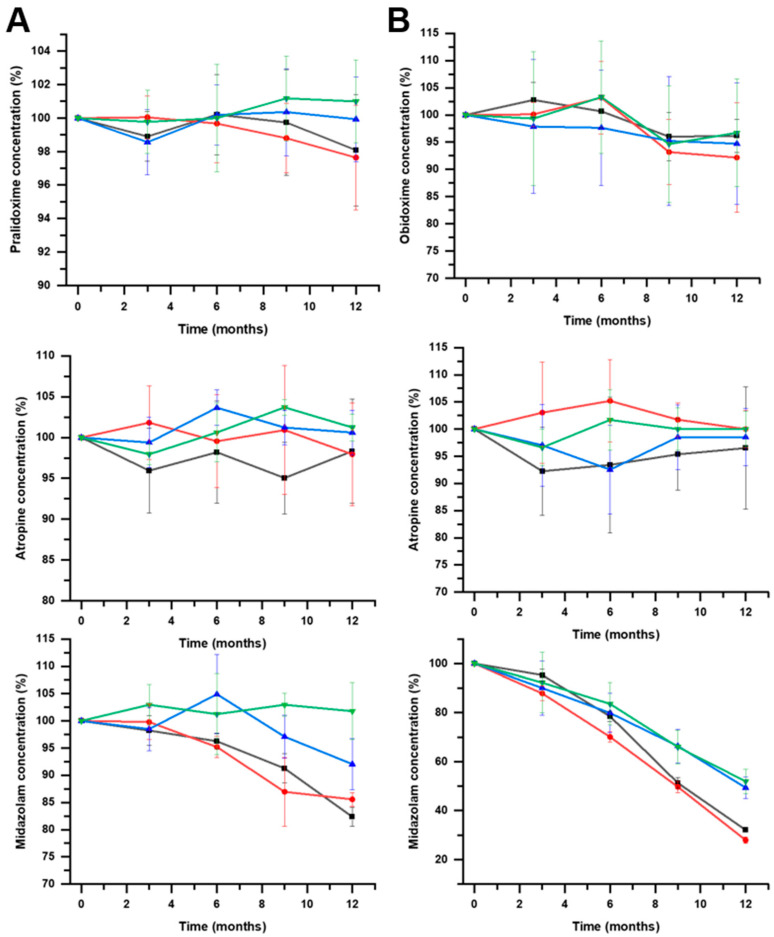
Mean results (n = 3) and standard deviation for pralidoxime, obidoxime, atropine, and midazolam within F1 (panel **A**) and F2 (panel **B**) formulations stored at 4 ± 2 °C. Key: pH3 JEA (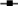), pH3 JEN (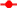), pH4 JEA (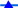), pH4 JEN (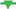).

Figure 4 shows the chemical stability of drug components of F1 and F2 at 4 °C. According to USP Pharmacopoeia (USP 38 (2015)), the lower specification limits are 90% for pralidoxime, obidoxime, and midazolam, while the lower specification limit for atropine is 93% [17]. In Table 2, the t_90_ and t_93_ values for samples stored at 25 ± 2 °C are illustrated. 

**Figure 5:**
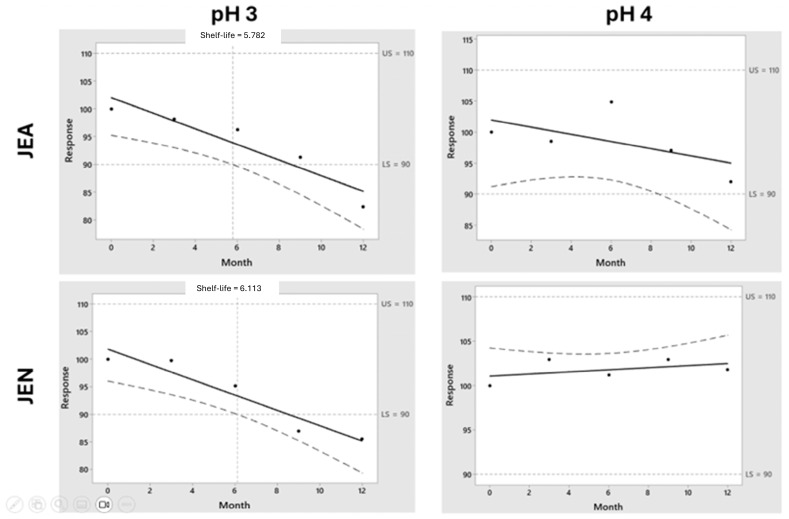
Shelf-life prediction for midazolam in F1 formulation at 4 °C. Key: The black dots represent the experimental points while the black solid line represents the predicted drug degradation kinetic from which the shelf-life from each formulation can be extrapolated, being intended as the time in which there is 95% confidence that at least 50% of response is within the specification limits. An upper limit and lower limit of 110 and 90% (dotted line) were taken into consideration for pralidoxime, obidoxime, and midazolam, while 107–93% was considered for atropine.

**Figure 6:**
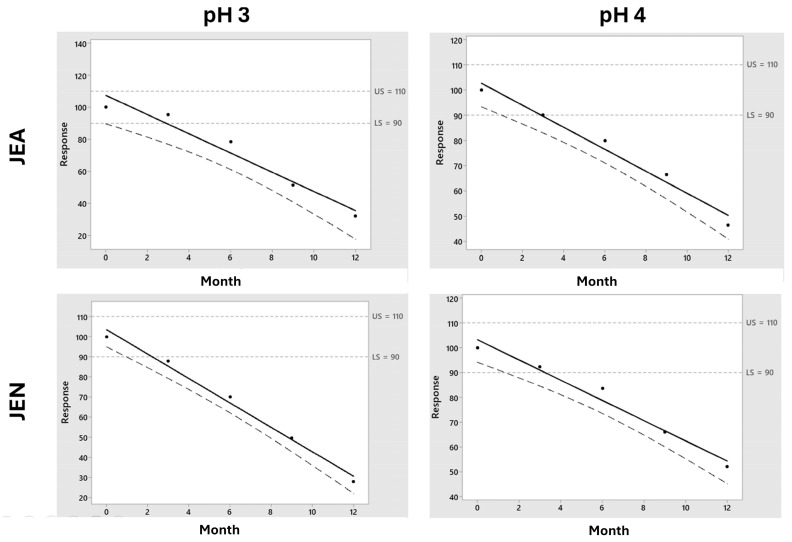
Shelf-life prediction for midazolam in F2 formulation at 4 °C. Key: The black dots represent the experimental points while the black solid line represents the predicted drug degradation kinetic from which the shelf-life from each formulation can be extrapolated, being intended as the time in which there is 95% confidence that at least 50% of response is within the specification limits. An upper limit and lower limit of 110 and 90% (dotted line) were taken into consideration for pralidoxime, obidoxime, and midazolam while 107–93% was considered for atropine.

Figure 4 shows the importance of the stored temperature on the chemical stability of midazolam. Pralidoxime, obidoxime, and atropine showed stability above 12 months when stored at 4 °C. It is worth highlighting the fast degradation kinetics of midazolam in both F1 and F2 formulations, which are accentuated in the F2 formulation at pH 4. In our experimental conditions, midazolam is incompatible with the rest of the components of the studied formulations. The shelf-life prediction for midazolam at 4 °C in F1 at pH 3 was 5.8 and 6.1 months when stored using JEA and JEN, respectively, while it was superior at 12 months at pH 4 (Figure 5). However, the chemical stability of midazolam at 4 °C in F2 was below 3 months in all the tested conditions (Figure 6). This shows the physicochemical interaction between oximes being more accentuated between obidoxime and midazolam in F2, which accelerates the degradation kinetics of midazolam. 

**Figure 7:**
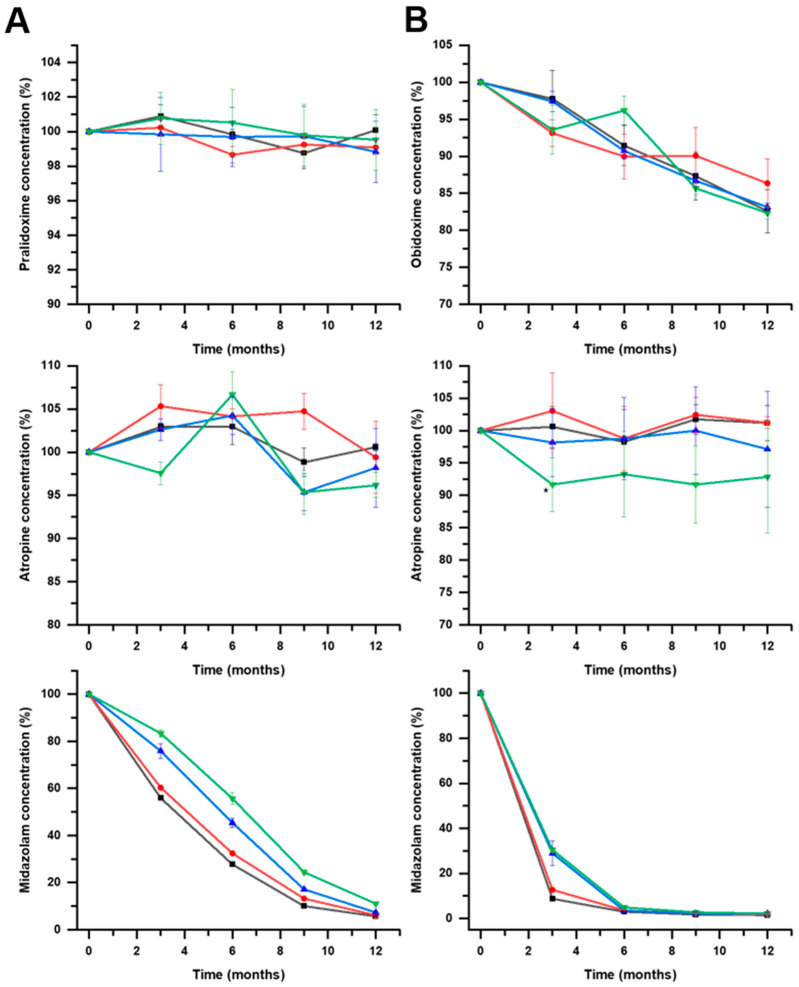
Mean results (n = 3) and standard deviation for pralidoxime, obidoxime, atropine, and midazolam within F1 (panel **A**) and F2 (panel **B**) formulations stored at 25 °C. Key: pH3 JEA (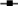), pH3 JEN (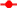), pH4 JEA (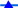), pH4 JEN (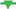).

Figure 7 shows the stability of all components for the F1 and F2 formulations at 25 °C. The t_90_ midazolam values at 25 ± 2 °C were lower than 1 month for F1 in all tested conditions and below 2 months for F2 formulations. The stability of autoinjector monocomponent formulations containing only midazolam stored at 25 °C in similar conditions as those described in this work was about two years [14]. This shows evidence of the effect of temperature on accelerating the degradation kinetics of midazolam. A possible practical solution to alleviate this problem is to prepare autoinjectors with two different compartments to avoid the interactions between midazolam and the other two API components. 

**Figure 8:**
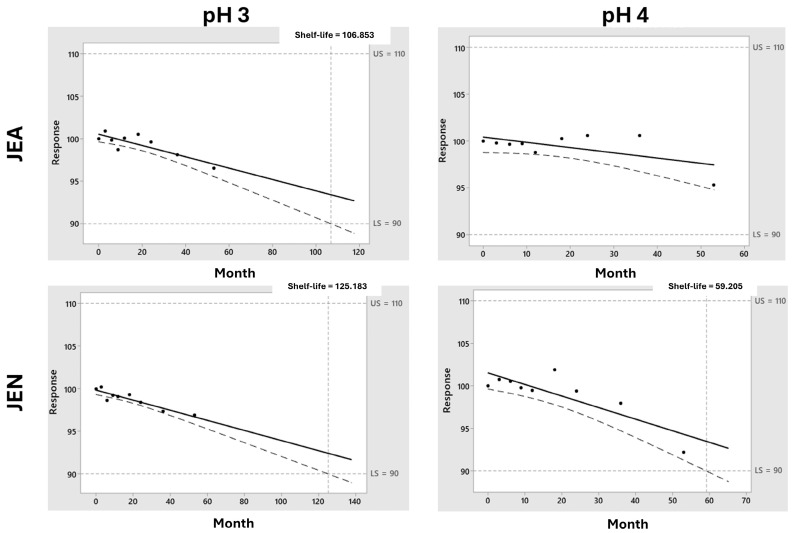
Shelf-life prediction for pralidoxime in F1 formulation at 25 °C. Key: The black dots represent the experimental points while the black solid line represents the predicted drug degradation kinetic from which the shelf-life from each formulation can be extrapolated, being intended as the time in which there is 95% confidence that at least 50% of response is within the specification limits. An upper limit and lower limit of 110 and 90% (dotted line) were taken into consideration for pralidoxime, obidoxime, and midazolam while 107–93% was considered for atropine.

**Figure 9:**
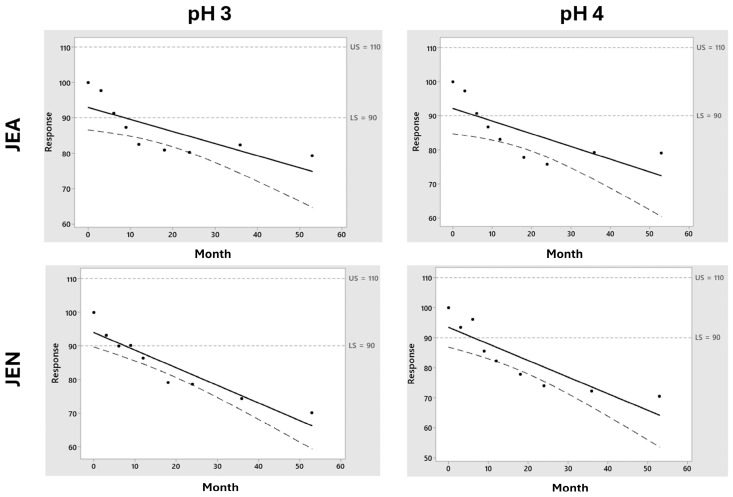
Shelf-life prediction for obidoxime in F2 formulation at 25 °C. Key: The black dots represent the experimental points while the black solid line represents the predicted drug degradation kinetic from which the shelf-life from each formulation can be extrapolated, being intended as the time in which there is 95% confidence that at least 50% of response is within the specification limits. An upper limit and lower limit of 110 and 90% (dotted line) were taken into consideration for pralidoxime, obidoxime, and midazolam while 107–93% was considered for atropine.

Atropine showed great stability (>5 years) in all the tested conditions in both the F1 and F2 formulations at 25 °C. However, between the oximes, significant differences were encountered. F1 formulations containing pralidoxime exhibited chemical stability above 100 months at pH 3 but lower at pH 4 (Figure 8), while obidoxime in F2 formulations showed lower stability below 12 months in all cases (Figure 9). Combinations of pralidoxime and atropine were stable for longer than 5 years when stored at 25 °C in all tested formulations. 

In previously reported studies [21], the combination of atropine and obidoxime stored at 25 °C showed good stability for two years. However, in our experimental conditions, the stability of obidoxime combinations with atropine stored at 25 °C was lower than one year. This detriment in the chemical stability of obidoxime can be linked to the physicochemical interaction with midazolam. 

**Figure 10:**
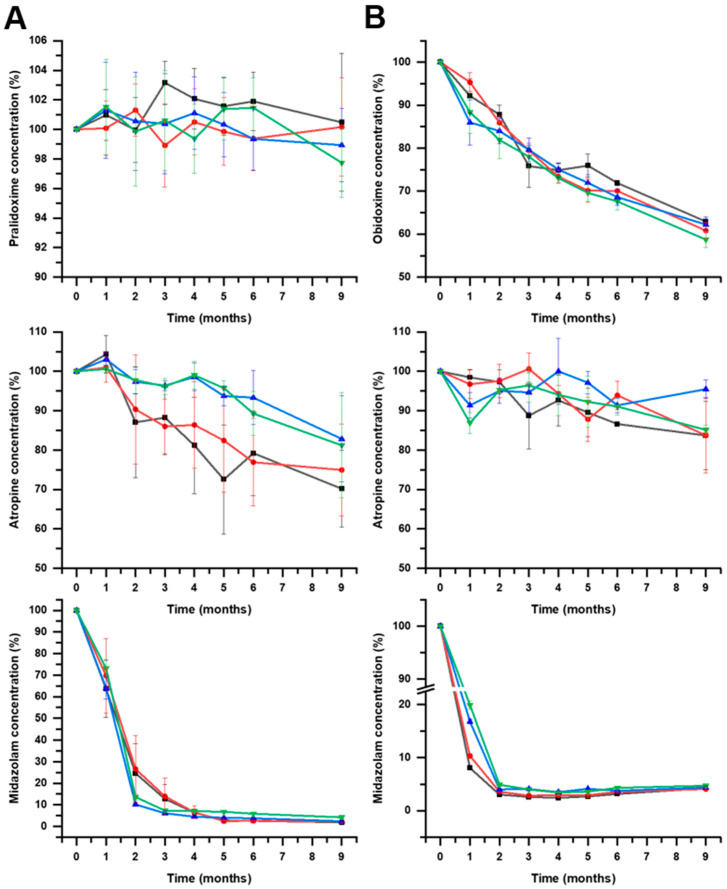
Mean results (n = 3) and standard deviation for pralidoxime, obidoxime, atropine, and midazolam within F1 (panel **A**) and F2 (panel **B**) formulations stored at 40 °C. Key: pH3 JEA (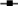), pH3 JEN (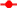), pH4 JEA (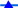), pH4 JEN (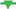).

The chemical stability of all drug components at 40 °C is illustrated in Figure 10. Pralidoxime showed optimal stability, even at higher temperatures compared to all other APIs. The midazolam and obidoxime degraded fast at 40 °C. The effects of pH and type of sealing material were highly dependent on the API component. For obidoxime, pH 3 improved the stability (*p* < 0.1) compared to pH 4. However, for atropine and midazolam, pH 4 was preferable (*p* < 0.1) to pH 3. In previous reported [13] studies, JEN showed better compatibility than JEA for midazolam. This work corroborates these results as JEN was preferable to JEA for midazolam (*p* < 0.1) and obidoxime (*p* < 0.1). The poor chemical stability of midazolam and obidoxime combined with other APIs could not be improved by changes in pH or the type of sealing to obtain a t_90_ longer than 1 year at 25 ± 2 °C. 

Table 2 shows the mean and standard deviation of API adsorption expressed as the percentage of the API content of F1 and F2 after 53 months at 25 °C. Adsorption was significantly (*p* < 0.1) related to the lipophilicity of the API for both studied sealing materials. However, there were significant differences between JEA and JEN (*p* < 0.1). JEN has a lower adsorption for the APIs than JEA. Therefore, it can be concluded that JEN bromobutyl gaskets were preferable to JEA to avoid drug adsorption.

## 4. Conclusions

The chemical stability of different multicomponent formulations, including an oxime (pralidoxime or obidoxime), atropine, and midazolam, was evaluated at different temperatures between 4 and 40 degrees. Pralidoxime showed a better stability profile compared to obidoxime. Midazolam was unstable in all the tested experimental conditions. Drug adsorption was observed in both types of sealing elastomeric materials and was significantly (*p* < 0.01) dependent on the lipophilicity of the drug. The bromobutyl gasket was a better alternative to minimize drug adsorption. It can be concluded that the most stable formulation consisted of pralidoxime and atropine at pH 4 with the elastomeric sealing material 4023/50 GRAY. 

## Figures and Tables

**Table 1:** Conditions of the stability study and quality parameters tested.

Study	Storage Conditions	Minimum Time According to ICH	Time of the Study	Testing Frequency (Months)	Quality Parameters Tested
Long term	25 ± 2 °C 60 ± 5% R.H.	12 months	53 months	0, 3, 6, 9, 12, 18, 24, 36 and 53	- APIs concentration - APIs adsorbed in the elastomeric material - Physical appearance of the liquid formulations and the elastomeric sealing material
Accelerated	40 ± 2 °C 75 ± 5% R.H.	6 months	9 months	1, 2, 3, 4, 5, 6, and 9	
Refrigerated	4 ± 2 °C	6 months	12 months	3, 6, 9, and 12	

**Table 2:** API content adsorbed on elastomeric sealing materials. Key: values represented correspond to the mean (n = 3) ± SD of API adsorbed in F1 and F2 samples stored at 25 °C for 53 months.

Formulation	Variables	Pralidoxime	Atropine	Midazolam
F1	pH 3 JEA	0.020 ± 0.001	0.038 ± 0.006	0.284 ± 0.003
	pH 3 JEN	0.021 ± 0.002	0.041 ± 0.011	0.170 ± 0.002
	pH 4 JEA	0.022 ± 0.001	0.066 ± 0.023	0.440 ± 0.003
	pH 4 JEN	0.014 ± 0.001	0.025 ± 0.010	0.409 ± 0.007
F2	pH 3 JEA	0.060 ± 0.002	0.039 ± 0.014	0.305 ± 0.045
	pH 3 JEN	0.023 ± 0.001	0.038 ± 0.006	0.310 ± 0.003
	pH 4 JEA	0.012 ± 0.001	0.032 ± 0.007	0.217 ± 0.005
	pH 4 JEN	0.017 ± 0.001	0.039 ± 0.002	0.203 ± 0.007

## Data Availability

Data is contained within the article.

## References

[1] John H., van der Schans M.J., Koller M., Spruit H.E.T., Worek F., Thiermann H., Noort D. (2018). Fatal sarin poisoning in Syria 2013: Forensic verification within an international laboratory network. Forensic Toxicol..

[2] Steindl D., Boehmerle W., Körner R., Praeger D., Haug M., Nee J., Schreiber A., Scheibe F., Demin K., Jacoby P. (2021). Novichok nerve agent poisoning. Lancet.

[3] Haslam J.D., Russell P., Hill S., Emmett S.R., Blain P.G. (2022). Chemical, biological, radiological, and nuclear mass casualty medicine: A review of lessons from the Salisbury and Amesbury Novichok nerve agent incidents. Br. J. Anaesth..

[4] Ziemba R. (2012). Use of individual auto-injector kits ‘IZAS-05’ on the contemporary battlefield. Med. Sci. Monit..

[5] Lallement G., Clarencon D., Brochier G., Baubichon D., Galonnier M., Blanchet G., Mestries J.C. (1997). Efficacy of Atropine/Pralidoxime/Diazepam or Atropine/HI-6/Prodiazepam in Primates Intoxicated by Soman. Pharmacol. Biochem. Behav..

[6] Clair P., Wiberg K., Granelli I., Carlsson Bratt I., Blanchet G. (2000). Stability study of a new antidote drug combination (Atropine-HI-6-Prodiazepam) for treatment of organophosphate poisoning. Eur. J. Pharm. Sci..

[7] RamaRao G., Afley P., Acharya J., Bhattacharya B.K. (2014). Efficacy of antidotes (midazolam, atropine and HI-6) on nerve agent induced molecular and neuropathological changes. BMC Neurosci..

[8] Bajgar J. (2012). Antidotal treatment. Nerve Agents Poisoning and Its Treatment in Schematic Figures and Tables.

[9] Schroeder A.C., DiGiovanni J.H., Von Bredow J., Heiffer M.H. (1989). Pralidoxime chloride stability-indicating assay and analysis of solution samples stored at room temperature for ten years. J. Pharm. Sci..

[10] Corvino T.F., Nahata M.C., Angelos M.G., Tschampel M.M., Morosco R.S., Zerkle J., Nelson R.N. (2006). Availability, stability, and sterility of pralidoxime for mass casualty use. Ann. Emerg. Med..

[11] Rubnov S., Shats I., Levy D., Amisar S., Schneider H. (1999). Autocatalytic degradation and stability of obidoxime. J. Pharm. Pharmacol..

[12] Berton B., Chennell P., Yessaad M., Bouattour Y., Jouannet M., Wasiak M., Sautou V. (2020). Stability of Ophthalmic Atropine Solutions for Child Myopia Control. Pharmaceutics.

[13] Gilliot S., Masse M., Feutry F., Barthélémy C., Décaudin B., Genay S., Odou P. (2020). Long-term stability of ready-to-use 1-mg/mL midazolam solution. Am. J. Health Syst. Pharm..

[14] Rodríguez Fernández M.J., Serrano Lopez D.R., Torrado J.J. (2022). Effect of Primary Packaging Material on the Stability Characteristics of Diazepam and Midazolam Parenteral Formulations. Pharmaceutics.

[15] Reichard D.W., Atkinson A.J., Hong S.P., Burback B.L., Corwin M.J., Johnson J.D. (2010). Human safety and pharmacokinetic study of intramuscular midazolam administered by autoinjector. J. Clin. Pharmacol..

[16] ICH (2003). Stability Testing of New Drug Substances and Products Q1A(R2).

[17] (2015). Pharmacopoeia-National Formulary.

[18] Ghosh S., Chatterjee M., Roy K. (2023). Predictive Quantitative Read-Across Structure-Property Relationship Modeling of the Retention Time (Log tR) of Pesticide Residues Present in Foods and Vegetables. J. Agric. Food Chem..

[19] Torrado-Salmeron C., Laguna A., Guillén A., Saro M.G., Matji A., Torrado J.J., Serrano D.R. (2022). Tailoring Rational Manufacturing of Extemporaneous Compounding Oral Dosage Formulations with a Low Dose of Minoxidil. Pharmaceutics.

[20] González-González O., Ballesteros M.P., Torrado J.J., Serrano D.R. (2023). Application of Accelerated Predictive Stability Studies in Extemporaneously Compounded Formulations of Chlorhexidine to Assess the Shelf Life. Molecules.

[21] Ettehadi H.A., Ghalandari R., Shafaati A., Foroutan S.M. (2013). Development of a combined solution formulation of atropine sulfate and obidoxime chloride for autoinjector and evaluation of its stability. Iran J. Pharm. Res..

